# Therapeutical and Pharmaceutical Notes

**Published:** 1901-08

**Authors:** W. J. Martin

**Affiliations:** Kankakee, Ill.


### THERAPEUTICAL AND PHARMACEUTICAL NOTES.

UNDER THE DIRECTION OF W. J. MARTIN, M.D.C.,
KANKAKEE, ILL.

---

DIGITALIS AND ITS ACTIVE CONSTITUENTS. Digitalis, popularly known as foxglove, is a native plant of the mountainous regions of all western Europe; it is found from Portugal to Finland and from middle Germany down to Italy. Its medicinal virtues do not seem to have been known to the ancient writers of Greece and Rome, Northern people appearing to have first discovered its remedial powers; but its use originally was only for external purposes, as is evidenced by a treatise of the thirteenth century on the practice of medicine in Wales, Great Britain. Indeed, its value was long doubted by physicians, for as late as in the last quarter of the eighteenth century Murry speaks of foxglove as a doubtful remedy; but in the year 1775 Withering published his experience with this plant in the treatment of dropsy, which may be looked upon as the beginning of its career in scientific medicine. Through Thileus digitalis became known to the Germans, whence its reputation began to spread over the rest of the Continent; but although this drug has been before the medical profession for over 350 years, and since at least 1775 has furnished the subject-matter for many voluminous essays, yet it may safely be asserted the majority of practitioners are as much in the dark regarding the physiological relations and rational administration of this potent drug as were their forefathers of several generations ago. In this connection a portion of a paper published not long ago by Dr. Archie Stockwell in the *Monthly Cyclopædia of Practical Medicine* may prove interesting reading. We quote:

*Pharmacology of Digitalis.* Up to within a quarter of a century digitalis was commonly described as an adenalgic, antiphlogistic, antipyretic, and heart sedative, but chiefly commended as a diuretic, though admittedly possessed of considerable unreliability. Indeed, so much was said and claimed for the drug that the expectations of the newly fledged practitioner were placed entirely too high, for theoretical assumption and speculation generally usurped the place of rational, careful, and practical study; and even to-day, though modern research seems to have conclusively established that digitalis is not a cardiac sedative and depressant, but, instead, a tonic and stimulant, this knowledge accrues less to a better understanding of the drug than to fuller comprehension of physiological problems connected with the circulatory apparatus. There are many who yet hold to sedation of the heart by digitalis, and the agent is still prescribed in a most empirical way for all forms and classes of cardiac and arterial disease from syncope and simple hypertrophy up to aneurism and exophthalmic goitre.

The actions of digitalis are varied and multiple, being materially modified by many causes, such as dosage, temperature, humidity, and other climatic surroundings; presence or absence of the febrile state; condition of excretion; idiosyncrasy (and there is no remedial agent toward which persons are more generally idiosyncratic, oftentimes unaccountably so, and in varying degrees at different periods). Again, the drug often has an elective affinity, so to speak, for certain organs and tissues, according to the character of the preparation employed; notably, too, stimulant action upon the heart and circulation may not be made manifest until twenty-four or thirty-six hours after the initial dose; while, on the other hand, the same action may persist from three to seven days after the medicament has been withdrawn.

Sometimes supposed idiosyncrasy may lie wholly with the prescriber, owing to ignorance of the character of the preparation employed and the misconception of "affinities," both physiological and pharmacological. Every practitioner should realize that the average tincture as had in the shops is usually inert, being, on the score of cheapness, derived from dried and pressed leaves of uncertain age, plucked, perhaps, at any season and stage of the plant's growth, and cured without method. It is well understood by pharmacologists that little or no virtues accrue to foliage that is not of the second year of growth of the uncultivated foxglove, which must be gathered just before the close of the flowering season, and especially dried in the dark at a carefully regulated temperature; also, that the best digitalis, after ten or twelve months, even if kept in well-guarded and stoppered tins and jars, will have parted with most of its virtues. Hence, foreign pharmacopœias lay particular stress upon selecting freshly-gathered leaves, especially for preparing infusions, tinctures, and fluid extracts. Adulteration is another source of uncertainty and annoyance, the leaves of the black nightshade, black mullein, and even of the common potato, or all three, being utilized for this purpose, though fortunately all are easily detected by making a concentrated infusion with a suspected leaf and testing on an opalescent plate by means of a drop of ferric chloride. If the reaction is a deep green, the leaf is of the foxglove ; if blue, of an adulterant.

*Relative Strength of Preparations.* Tinctures made with the aid of fluid extracts do not by any means represent the properties of the drug in the same degree as those had by means of maceration and percolation of the freshly dried and gathered leaves. Neither do the solid and fluid extracts, or abstracts, unless made by the substitution process and with the avoidance of any but the most gentle heat, represent the true virtues of digitalis. A brown or black hue to the exclusion of green color is always *prima facie* evidence of more or less improper manufacture. The tinctures of the *British Pharmacopœia* and *United States Pharmacopœia* vary slightly as to the amount and strength of contained spirit, while the proportions of the drug are, respectively, 3 to 24, and 3 to 20. Notably, the most reliable tinctures are those had from German, eclectic, or homœopathic sources, all of which are made from fresh, undried leaves. The two former by maceration and percolation, the latter by expression of juice and subsequent mingling, with an equal proportion by weight of 87° alcohol. Two other excellent preparations that meet with favor in Europe are the ethereal tincture—which is twice the strength of the alcoholic tincture of the *United States Pharmacopœia*—and acetum digitalis, made by macerating one ounce of the leaves in nine ounces of vinegar and one ounce of alcohol. Tablets, or tablet triturates, made with the so-called "concentration" digitalisin, are dangerous, since this preparation is by no means constant as to strength. Tablets, also purporting to embody the tincture or fluid extract of the drug, are, for obvious reasons, usually inert, and hence not to be trusted.

Latterly, the so-called "active principles" have been widely exploited, chiefly to the benefit of manufacturers and dealers, but none that are deemed unrepresentative or inert are of a character to deserve confidence, at least not until more precise knowledge accrues thereto. All are glucosides and not alkaloids, as has been assumed, and notoriously are not constant as to either therapeutic or physical properties, being capable of further chemical subdivision. Thus digitoxin, which has been deemed most reliable, because it appears as a component constituent of all except digitonin and digitin (the latter physiologically inert), yields digitoxiresin and a body as yet not fully identified, and consequently unnamed. We can only re-echo the opinion of Roth, that digitalin, digitalein, digitaline, digitaleine, digitalinum verum, digitoxin, digitaliresin, and digitoxiresin "are not to be recommended," since they are in dosage and title, and often the same (supposed) precise agent is greatly at variance as regards different samples.

One of the greatest bugbears accruing to digitalis is its supposed "cumulative action," echoed and re-echoed for more than a century, and which has led to over-cautious use, even at times when administration was demanded boldly and in large doses. In truth, there is no such thing as "cumulative action," except in a way any drug may become cumulative, owing to ignorance or carelessness on the part of the prescriber, tending to a disregard of the problems of elimination. When the blood-pressure is at its height the functions of excretion as carried on by the kidneys may be arrested, and likewise the functions of the skin, and it is to the inhibition of elimination that any untoward ("cumulative") action is due, and even this may often be prevented by exhibiting the drug in conjunction with some more direct stimulant to the renal organs.

*Dosology of Digitalis.* That digitalis is not the highly dangerous remedy surmised the common everyday literature of the drug fully attests. The late Dr. Jones, of the Island of Jersey, was wont to give the tincture in four-drachm doses, repeated every fourth hour, in delirium tremens, and thereby successfully treated some seventy cases without a single untoward result, and scores of practitioners have borne evidence as to the value and utility of the method, including Ringer and Sainsbury, as recently as last year. Dr. King, of Suffolk, England, was accustomed to administer like doses for the purpose of combating acute inflammation. He sometimes gave two drachms of tincture to children under twelve months old, and in all his extensive experience never witnessed a single dangerous symptom. Pereira declares he saw twenty drops of tincture prescribed three times daily for a fortnight to an infant suffering from hydrocephalus, and that he himself frequently gave a drachm of "the best quality" to an adult thrice daily for two weeks or more "without observing any marked effects." And, finally, H. C. Wood asserts that during an experience of twenty-five years, often with very large doses, he "never saw a case where digitalis seemed to do serious harm by a toxic action."

Unfortunately, a large share of the information that accrues to digitalis is of a speculative or presumptive character, being based upon experiments conducted through the medium of small mammals, birds, and batrachians; but what reliance can be placed upon evidence derived from procedures involving guinea-pigs, rabbits or hares, frogs, etc.? The first two are possessed of exceedingly sensitive nervous organisms, and their timidity has passed into proverbs and wise saws; they often die under manipulation, purely from fright (it may be here remarked that the most trifling shock may prove fatal, as when shot or snared, and they do not die as the result of the wound or noose, but of ruptured hearts).[1]

Experiments on birds and frogs may be even more misleading than those on mammals. There are no parallels between them and the human subject as to food, physiological processes, habits of life, or elimination of waste. The bird presents a stomach practically devoid of absorptive powers, and the digestive functions are adapted to the forms of food; it also has a three-chambered instead of a four-chambered heart, an anatomical peculiarity that invariably carries with it the excretion of solid urine. More remarkable are the relations of the two-chambered hearts of the so-called cold-blooded batrachians and reptiles, but this phase of the question will not be discussed in detail now.

Thus far, Dr. Stockwell.

For comparison we add what Dr. Hobart A. Hare has written in the *Therapeutic Gazette* on the choice of the various preparations of digitalis. He says:

"For many years members of the medical profession have been wont to regard the various pharmaceutical preparations of digitalis as possessed of a widely different physiological effect, far exceeding the variation naturally arising from the relative strength of an infusion as compared to a tincture. In other words, it has been generally conceded that no dose, large or small, of the infusion could be hit upon which would produce effects caused by any dose of the tincture; or, to express it still more clearly, it has been the general view that each of these preparations was capable of producing effects peculiar in some respects to itself. It has been supposed that this variation depends upon the relative proportions

[1] Of twenty rabbits selected at random in market that had been shot seventeen exhibited wounds that could not in any way account directly for death, and the seventeen all had ruptured left ventricles. A single shot-pellet that penetrates the skin barely enough to produce slight ecchymoses of the tissues beneath is often fatal to the hare, and when the creature is snared there is often evidence that the wire had not performed its work when death occurred, and here inevitably the heart ventricle is found torn. A cart-horse in Dumfries, Scotland, seven years old, fell dead on viewing for the first time a railway engine, and post-mortem revealed a ruptured heart, with no trace of disease anywhere.

of the various active principles of digitalis held in solution by the water or alcohol with which the preparation has been made, and if it be true that each preparation has an effect of its own, it is also undoubtedly true that this is due to the reasons just suggested, as I am about to point out.''

*Active Principles of Digitalis.* It will be recalled that digitalis contains at least five principles, of which four are physiologically active and the fifth inactive. From these there may be developed other substances by chemical alterations or decompositions, but they probably are not primarily present. Each of these ingredients possesses a physiological action of its own, and each has a solubility of its own. Of the four active constituents, digitalin, digitoxin, and digitalein act upon the heart muscle, while digitonin has an entirely different effect, namely, the power of depressing the vagus nerves centrically and peripherally and the inhibitory ganglia in the heart. The digitalin here referred to is not the digitalin of amorphous form prepared according to the process of Homolle, nor the crystalline digitalin of Nativelle, neither of which is a pure digitalin, but it is the digitalin of Schmiedeberg.

The effect of Schmiedeberg's digitalin upon the heart is that of a powerful stimulant, for under its influence the individual heart-beats become more powerful (four to six times greater than normal); and it simultaneously causes a rise of blood-pressure, first, by increasing the strength of the heart, and, second, by stimulating the centric and peripheral vasomotor apparatus.

The physiological effects of digitalein and digitoxin are identical with those of digitalin, except that they do not stimulate the vasomotor centre nor the pneumogastric apparatus, and so do not directly raise blood-pressure or slow the heart; in other words, they increase the force of ventricular contraction. The effect of digitonin being to depress the vagus nerves, it will be seen at once that it antagonizes the vagal effect of the digitalin on these fibres, and so prevents digitalis from slowing the heart to the extent that would result from the use of digitalin alone. It also depresses the heart muscle. The proportion of digitonin in digitalis varies, but it is not present in sufficient amount to entirely overcome the inhibitory influence of the digitalin.

If we now turn to a consideration of the solubilities of these principles we can readily explain the different effects produced by the infusion and tincture and fluid extract. Digitonin is soluble in water, as is digitalein, but digitalin is only slightly soluble, and digitoxin is scarcely soluble in water at all. As a result, the use of the infusion in a case of heart disease would not give the patient the same degree of cardiac power as the use of the tincture, for not only would the most powerful stimulant of all to the heart, vasomotor system, and vagi be present in small amount, but in addition the large proportion of digitonin would antidote it.

On the other hand, digitonin is sparingly soluble in alcohol, while digitalin and digitalein are readily soluble in it, digitoxin being slightly so. It would seem, therefore, that in the presence of a failing heart and circulation the tincture or the fluid extracts are the preparations greatly to be preferred to the infusion, because they contain large amounts of the active stimulant ingredients.

The reason that the infusion acts efficiently in some cases as a diuretic probably depends upon the fact that as it does not contain so much digitalin it is less apt to cause spasm of the renal vessels, but if the heart is feeble and there is renal stasis the tincture is probably the better preparation to overcome this state, because it both aids the heart and, by contracting the renal vessels, overcomes the stasis.

The use of digitalin is inadvisable unless we are sure that we get that made according to the process of Schmiedeberg, for the other digitalins usually sold are very uncertain. The infusion is far more apt to disorder the stomach than the fluid extract or tincture, because of the irritating digitonin.—*Western Druggist.*

